# CCT2 enhances c-Myc stabilization to drive tumor progression and chemoresistance in head and neck squamous cell carcinoma

**DOI:** 10.1016/j.gendis.2025.101795

**Published:** 2025-08-07

**Authors:** Lin Lu, Yi Shen, Xuerong Li, Yiwei Zhao, Xuefan Zhai, Min Cai, Baicheng Bao, Guiqing Liao, Jianbo Sun

**Affiliations:** a Hospital of Stomatology, Guanghua School of Stomatology, Sun Yat-Sen University, Guangzhou, Guangdong 510055, China; b Guangdong Provincial Key Laboratory of Stomatology, Guangzhou, Guangdong 510055, China; c Dongguan Key Laboratory of Chronic Inflammatory Diseases, The First Dongguan Affiliated Hospital, Guangdong Medical University, Dongguan, Guangdong 523710, China

**Keywords:** Apoptotic vesicles, c-Myc, CCT2, Cisplatin resistance, Head and neck squamous cell carcinoma

## Abstract

CCT2, a vital subunit of the chaperonin-containing TCP-1 (CCT) complex, has emerged as a critical regulator of protein homeostasis and cancer progression. In this study, we highlight the essential role of CCT2 in head and neck squamous cell carcinoma (HNSCC), where it is overexpressed and strongly associated with poor prognosis, advanced clinical stages, and aggressive tumor behavior. Mechanistically, CCT2 interacts with and stabilizes the oncogenic c-Myc protein to drive tumor cell proliferation, migration, and invasion. Furthermore, CCT2 contributes to cisplatin resistance by regulating c-Myc-dependent pathways and cell cycle-related proteins. Notably, apoptotic vesicles derived from drug-resistant tumor cells transfer CCT2 to neighboring cells, further enhancing chemoresistance. These findings position CCT2 as a key driver of HNSCC progression and c-Myc-mediated oncogenic pathways, presenting it as a promising therapeutic target for overcoming drug resistance and improving treatment outcomes in HNSCC patients.

## Introduction

Head and neck squamous cell carcinoma (HNSCC) is a highly malignant cancer that arises from the epithelial cells of the oral cavity, pharynx, and larynx. Despite significant advancements in multimodal therapies, the prognosis for patients with HNSCC remains disheartening, and the 5-year survival rate is only around 60%.^1^ A major challenge in the treatment of HNSCC is its rapid progression during therapy, often accompanied by the development of cisplatin resistance,^2^ which significantly compromises therapeutic efficacy and contributes to recurrence and metastasis, ultimately leading to treatment failure.^3^ Understanding resistance mechanisms is key to developing new treatments and improving patient outcomes.

Chaperonin containing TCP-1 (CCT), also referred to as TRiC or c-cpn, belongs to the eukaryotic group II chaperonin family and consists of eight homologous subunits (CCT1–8). CCT interacts with 5%–10% of the cellular proteome and plays crucial roles in cell growth, proliferation, and apoptosis.^4^ It also interacts with several key oncogenic proteins, such as p53,^5^ Kirsten rat sarcoma viral oncogene homolog (KRAS),^6^ and signal transducer and activator of transcription 3 (STAT3).^7^ Emerging evidence underscores the pivotal role of CCT and its subunits in various malignancies, including breast,^8^ liver,^9^ gastrointestinal,^10^ lung cancer,^11^ as well as acute myeloid leukemia.^12^ Dysregulated expression of certain CCT family members has been linked to poor prognostic outcomes in these cancers. Among these subunits, CCT2 has drawn attention for its unique role in maintaining protein homeostasis, where it not only assists in the proper folding of proteins as part of the chaperonin complex but also functions as a monomer to mediate the clearance of protein aggregates.^13^ CCT2 is crucial for maintaining stemness in epithelial ovarian cancer and inducing drug resistance.^14^ It also promotes glioblastoma resistance to DHA by stabilizing KRAS,^6^ but further research is needed. c-Myc, an oncogene regulating 15% of the human genome, is frequently activated in cancers^15^ and promotes tumor progression, metastasis, and therapy resistance by dysregulating cell cycle, metabolism, and survival pathways,^16^, ^17^, ^18^ but targeting it presents challenges for the lack of specific binding sites and its nuclear localization.^19^ Targeting proteins that stabilize c-Myc could be a novel therapeutic strategy. However, the precise role of CCT2 in HNSCC and the related molecular mechanisms remain to be further investigated.

Additionally, chemotherapy-induced apoptosis generates abundant apoptotic vesicles (apoVs),^20^ which were once regarded as cellular debris for macrophage clearance.^21^ However, recent studies reveal that apoVs can deliver functional molecules, such as splicing factors to promote glioblastoma metastasis^22^ or aldehyde dehydrogenase (ALDH) to enhance stemness and chemoresistance in lung adenocarcinoma.^23^ Although CCT subunits have been reported to be enriched in glioblastoma-derived exosomes,^24^ whether CCT2 can mediate drug resistance through chemotherapy-induced apoVs remains to be investigated.

Our study showed here that CCT2 was highly expressed in HNSCC and was associated with poor prognosis. CCT2 promoted HNSCC cell proliferation and migration and reduced cisplatin sensitivity by stabilizing c-Myc. These findings suggested CCT2 as a viable therapeutic target to combat cisplatin resistance and improve the clinical outcomes of HNSCC, warranting further exploration.

## Materials and methods

### Public database analysis

RNA sequencing data and clinical information for HNSCC were obtained from the TCGA-HNSC dataset and four GEO datasets (GSE30784, GSE37991, GSE10121, and GSE127165). Tumor and adjacent normal tissue samples were analyzed to identify differentially expressed genes (DEGs). DESeq2 was used for TCGA data, while GEO2R was applied to the GEO datasets for DEG identification. Genes with a false discovery rate-adjusted *p*-value < 0.001 were considered significant, and those consistently overexpressed in tumor tissues were selected. The DEGs from all five datasets were intersected to identify genes that were consistently up-regulated in tumor tissues across datasets.

To further evaluate the prognostic significance of the 311 genes identified as significantly up-regulated in tumor tissues, univariate Cox regression analysis was performed using the GSE41613 and GSE42743 datasets. Genes with high expression significantly associated with poor prognosis (hazard ratio > 1) were identified, resulting in 129 and 79 genes, respectively. The intersection of these two gene sets yielded 45 genes. A forest plot was generated using R to visualize the hazard ratios and 95% confidence intervals of these 45 genes in the GSE41613 dataset. Survival analysis for CCT2 expression was performed in the GSE41613, GSE42743, and TCGA-HNSC datasets. Patients were categorized into high- and low-expression groups based on the optimal cutoff value, which was determined using the surv_cutpoint function from the survminer R package. Kaplan–Meier survival curves were constructed, and differences in survival were evaluated using the log-rank test.

In the TCGA-HNSC dataset, tumor samples were divided into high- and low-CCT2-expression groups based on the median expression level of CCT2. Gene Set Enrichment Analysis (GSEA) was performed to identify signaling pathways significantly enriched between the two groups. The analysis was conducted using the GSEA software, with pathways derived from Hallmark gene sets, and statistical significance was determined based on normalized enrichment scores and a false discovery rate < 0.05.

The 740 proteins interacting with CCT2 were retrieved from the BioGRID database. Enrichment analysis of these proteins was performed using the clusterProfiler R package, based on the Hallmark gene sets. The results of the enrichment analysis were visualized using a cnetplot, which illustrates the relationship between enriched pathways and the interacting proteins. The statistical significance of the enriched pathways was determined using an adjusted *p*-value < 0.05.

### Patients and follow-up

A total of 41 HNSCC patients who underwent surgery at Sun Yat-sen University's Guanghua School of Stomatology (Guangzhou, China) between January and December 2017 were included. None had prior radiotherapy or chemotherapy. Tissue samples from the primary tumor and adjacent peritumoral areas were collected for immunohistochemical analysis, confirmed by pathology. Informed consent was obtained from all participants, and clinical data were reviewed. Patients with death, recurrence, or metastasis were classified as poor prognosis, while others had a good prognosis. The study was approved by the Ethics Committee of Guanghua School of Stomatology, Sun Yat-sen University (Approval No. KQEC-2021-76-01).

### Immunohistochemistry (IHC)

Tissue sections from paraffin-embedded HNSCC samples or xenografts were deparaffinized, rehydrated, and treated with Triton X-100 and hydrogen peroxide. Antigen retrieval was done in sodium citrate buffer (pH = 6.0), followed by blocking with goat serum. Sections were incubated overnight with primary antibodies against CCT2 or Ki-67, and then with a secondary antibody. Staining was visualized using DAB (3,3-diaminobenzidine) and counterstained with hematoxylin. CCT2 or Ki-67 expression was evaluated by two pathologists, with staining scores < 25% considered negative (“−") and ≥25% considered positive (“+" for 25%–50%, "++" for 50%–75%, and "+++" for > 75%).

### Cell culture and transfection

Human HNSCC cell lines CAL-27 (ATCC), HSC-3 (Fuheng Biology), MOC1 (Fuheng Biology), SAS (Fuheng Biology), HOEC (Yaji Biology), and HIEC6 (Fuheng Biology) were mycoplasma-free and cultured as recommended. SAS and HSC-3 cells were transfected with PLVX-c-Myc-Neo plasmid (1 μg/well) using Lipofectamine 3000 and selected with 300 μg/mL G418 for two weeks. CCT2 or c-Myc knockdown was achieved using pLKO.1-TRC-puromycin-shRNA-CCT2 (shRNA1: TTATCGAGGAAGTCATGATTG; shRNA2: GCCTCTCTTATGGTAACCAAT), pLKO.1-TRC-puromycin-shRNA-MYC (shRNA1: CAGTTGAAACACAAACTTGAA; shRNA2: CCTGAGACAGATCAGCAACAA), and shRNA vectors, followed by selection in 3 μg/mL puromycin. Transfection efficiency was confirmed by quantitative PCR and Western blotting.

### Western blotting and quantitative PCR analysis

For total protein extraction, cells were lysed using RIPA buffer with protease and phosphatase inhibitors. Protein (20 μg) was separated by 10% SDS-PAGE, transferred to PVDF membranes, and blocked with 5% milk for 1 h. Membranes were incubated with primary antibodies at 4 °C overnight, followed by secondary antibodies at room temperature for 1 h. Protein bands were detected using the electrochemiluminescence-based sensing system. Primary antibodies are listed in Table S1. Original blots are shown in the Supplementary file.

Total RNA was extracted using TRIzol and reverse-transcribed into cDNA. PCR was performed using SYBR Green Master Mix Kit on a LightCycler 480 system. The results were normalized to GAPDH expression. PCR primers are listed in Table S2. These procedures were carried out as described in our previous study.^25^

### CCK-8, BrdU, and colony formation assays

shCCT2 and control cells were plated in 96-well plates at 3000 cells per well. At 24, 48, 72, and 96 h, 10 μL of CCK-8 reagent was added to each well, and absorbance at 450 nm was measured after 1 h.

For the BrdU incorporation assay, cells were treated with 10 μM BrdU (5′-bromo-2′-deoxyuridine) for 1–2 h, followed by fixation and permeabilization. BrdU-positive cells were detected using an anti-BrdU antibody, and DNA content was analyzed with 7-AAD (7-amino-actinomycin D) staining via flow cytometry.

For the colony formation assay, 1000 cells per well were seeded in 6-well plates and cultured for 10–14 days, with medium changed twice a week. Colonies were fixed with formaldehyde, stained with crystal violet, and manually counted if they contained more than 10 cells, as in a previous study.^25^

### Transwell and Boyden chamber assays

Cell migration and invasion were evaluated using Transwell assays. Following transfection, cells were seeded in the upper chambers of 24-well Transwell inserts (8 μm pore size) with serum-free medium, and the lower chambers contained complete medium with 10% fetal bovine serum. After 24–48 h, non-migratory cells were removed, and the migrated cells on the lower surface were fixed with paraformaldehyde and stained with crystal violet. For invasion assays, membranes were pre-coated with Matrigel. The migrated or invaded cells were captured in five random fields per well under a microscope.

### Wound healing assay

Cells were cultured in 6-well plates, scratched with a sterile pipette tip at 95% confluence, and washed with phosphate buffer saline solution (PBS). Serum-free medium was added, and wound closure was monitored over 24–48 h. Images of random fields were captured for analysis.

### 3D tumor spheroid migration assay

Cells were cultured to form 3D spheroids in low-attachment plates (Thermo Fisher Scientific). Mature spheroids were embedded in Matrigel (Thermo Fisher Scientific), and images were captured at 0 h and 72 h to assess migration. Spheroid expansion and cell dispersion were analyzed under a phase-contrast microscope (Carl Zeiss).

### Single-cell motility assay

Cells were seeded on flat-bottom 96-well plates, and live-cell imaging was conducted using an Operetta CLS high-content imaging system at 37 °C with 5% CO_2_. Images were captured every 30 min for 20 h. Single-cell migration speed (μm/s) was analyzed.

### TUNEL staining

Terminal deoxynucleotidyl transferase dUTP nick-end labeling (TUNEL) staining was performed to detect apoptotic cells using the manufacturer's instructions (Servicebio). Briefly, tissue sections or cultured cells were fixed with 4% paraformaldehyde and permeabilized with proteinase K. The sections were then incubated with the TUNEL reaction mixture containing terminal deoxynucleotidyl transferase and fluorescein-labeled nucleotides. After incubation, the sections were mounted with DAPI (4′,6-diamidino-2-phenylindole) to visualize the nuclei. Fluorescent signals were observed under a fluorescence microscope, and apoptotic cells were identified by the presence of green fluorescence.

### Cell apoptosis analysis

Cell apoptosis was assessed using a combination of 7-AAD and annexin V staining by flow cytometry. In brief, cells were collected and rinsed with PBS. For apoptosis analysis, cells were treated with annexin V-APC and 7-AAD following the manufacturer's protocol (BD Biosciences, California, USA).

### *In vivo* tumorigenesis

Xenograft experiments were approved by the Ethics Committee of the Hospital of Stomatology, Sun Yat-sen University (Approval No. SYSU-IACUC-2022-000027 and SYSU-IACUC-2020-000584), and adhered to 3Rs guidelines (replacement, reduction, refinement). Male BALB/c nude or C57BL/6 mice (8–10 weeks old) were randomly assigned to subgroups. A total of 5 × 10^6^ cells in 100 μL PBS were injected subcutaneously into the left flank. For C57BL/6 mice, cisplatin (5 mg/kg) was administered twice a week via intraperitoneal injection. Tumor growth was assessed every 3 days. Mice were anesthetized with 3% pentobarbital sodium (40 mg/kg) and euthanized on day 21 by cervical dislocation. Tumors were fixed in formalin, paraffin-embedded, and used for IHC analysis. Tumor volume was calculated using the formula: V = π/6 × (larger diameter) × (smaller diameter).^2^

### Co-immunoprecipitation

As stated before,^26^ co-immunoprecipitation was performed to assess the interaction between CCT2 and c-Myc. Cell lysates were prepared using immunoprecipitation lysis buffer containing protease inhibitors. For immunoprecipitation, the lysates were incubated at 4 °C overnight with an anti-CCT2 or anti-c-Myc antibody, followed by incubation with protein A/G agarose beads. The immunoprecipitated complexes were washed, eluted, and analyzed by Western blotting using antibodies against c-Myc and CCT2 to confirm their interaction.

### apoVs isolation and characterization

HSC-3 cells (control and shCCT2) were treated with 20 μM cisplatin for 48 h to induce apoptosis. After treatment, apoVs were isolated from the supernatant by sequential centrifugation: first at 800 *g* for 10 min to remove large debris, then at 2000 *g* for 10 min, and finally at 16,000 *g* for 30 min to collect the apoVs, which were resuspended in PBS for further analysis.

For nanoparticle tracking analysis, apoVs were diluted in filtered PBS, and particle size was measured using the ZetaView PMX120 system, with data processed by ZetaView software. Additionally, nanoflow cytometry (Flow NanoAnalyzer, NanoFCM Inc.) was used to assess particle concentration and size distribution, with samples adjusted to 4000–14,000 particles. apoVs were stained with annexin V-PE and anti-CCT2 antibodies, followed by secondary antibody staining.

Morphological analysis was done using transmission electron microscopy. apoVs were fixed in 1% glutaraldehyde, placed on copper grids, stained with uranyl acetate, and imaged using a JEM-1200EX TEM (JEOL). Immunofluorescence staining was performed to observe apoVs uptake. apoVs were labeled with PKH26 dye (Invitrogen) and incubated with anti-CCT2 antibodies, followed by secondary antibody staining. Nuclei were counterstained with DAPI, and images were captured using a Zeiss LSM 900 confocal microscope, as reported in a previous study.^27^

### Isolation of exosomes

When HSC-3 cells reached 80% confluency, the cells were rinsed with PBS, and the culture medium was replaced with fresh medium containing 5% exosome-depleted fetal bovine serum (Systembio). After 40 h, the culture supernatant was collected and centrifuged at 800 *g* for 10 min and 2000 *g* for 10 min to remove cellular debris. The resulting supernatant was then centrifuged at 16,000 *g* for 30 min to eliminate larger extracellular vesicles. Finally, the supernatant was ultracentrifuged at 120,000 *g* for 2 h to isolate the exosomes, as previously described.^27^

### Statistical analysis

Each experiment was performed in triplicate, and data were presented as mean ± standard deviation. Statistical comparisons between two groups were made using student's *t*-test, while multiple group comparisons were conducted using one-way ANOVA test. To compare the distribution of CCT2 IHC scores (ordinal variable: 0–3) between tumor and normal tissues, the Cochran-Armitage trend test (ordered chi-square test) was used to evaluate whether higher IHC scores were significantly associated with tumor tissues, assigning linear weights (0,1,2,3) to scores. Missing data were excluded. Pearson's correlation coefficient was calculated to evaluate relationships between two variables. A paired *t*-test was used to compare CCT2 expression in tumor and adjacent normal tissues from the same patient. Data analysis was carried out using SPSS 23.0 (IBM) and GraphPad Prism 9.1 (GraphPad Software). A *p*-value < 0.05 was considered statistically significant.

## Results

### CCT2 was up-regulated in HNSCC and associated with worse prognosis

**Figure 1:**
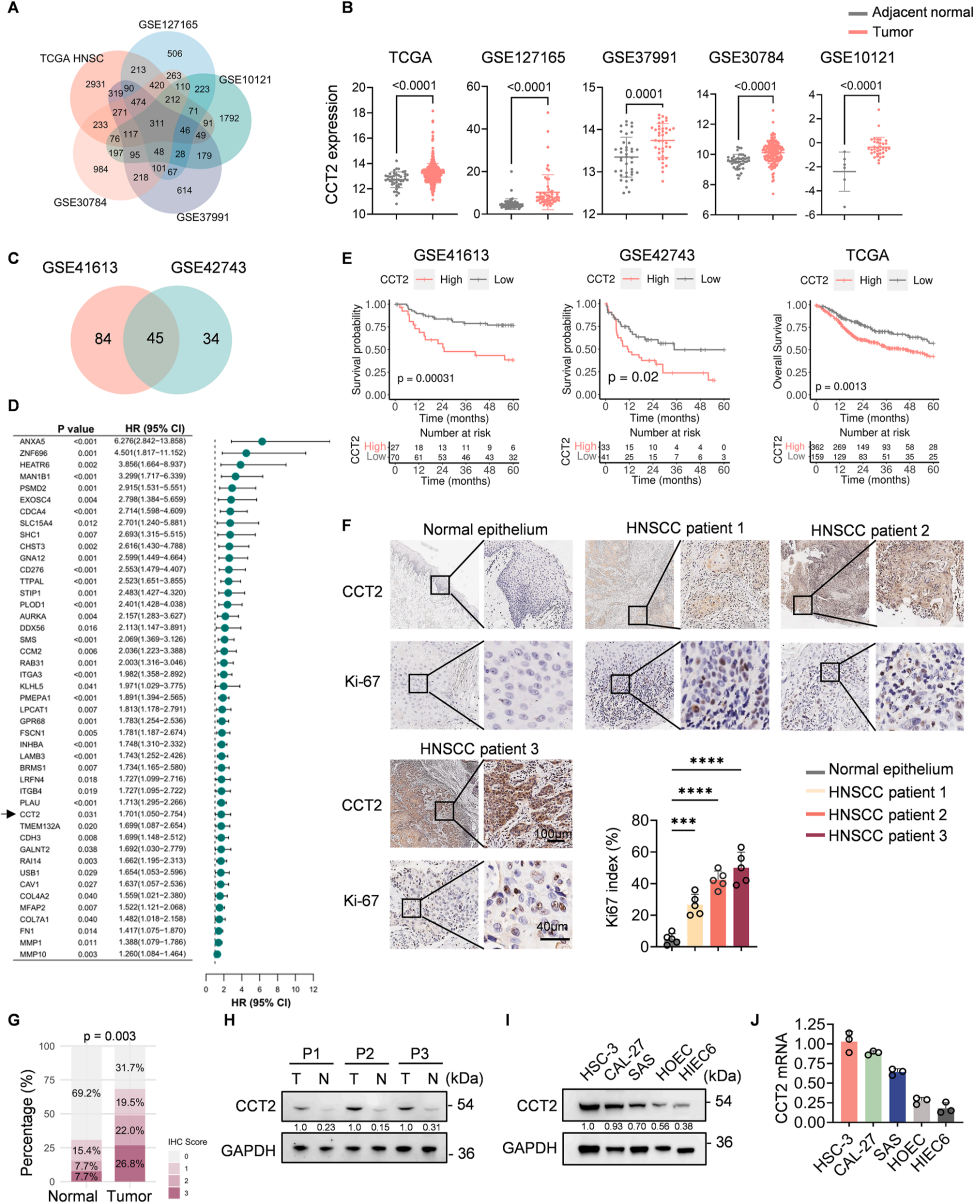
CCT2 was highly expressed in HNSCC and related to poor prognosis. **(A)** Up-regulation of 311 genes in HNSCC tumor tissues compared with adjacent normal tissues across the TCGA HNSC, GSE127165, GSE10121, GSE37991, and GSE30784 datasets. **(B)** CCT2 was highly expressed in tumor tissues compared with adjacent normal tissues. **(C)** Identification of 45 common prognosis-associated genes through univariate Cox regression analysis in the GSE41613 and GSE42743 datasets. **(D)** Forest plot of univariate Cox regression analysis for 45 common prognosis-associated genes in the GSE41613 dataset. **(E)** High expression of CCT2 predicted poor prognosis in GSE41613, GSE42743, and TCGA datasets. **(F)** Representative immunohistochemistry images illustrating the expression levels of CCT2 and Ki-67 in HNSCC tissues and normal epithelium. **(G)** Distribution of CCT2 immunohistochemistry scores in tumor and adjacent normal tissues. **(H)** Western blotting analysis of CCT2 expression was performed on paired tumor and adjacent normal tissues from three HNSCC samples. **(I, J)** Western blotting and quantitative PCR analysis of CCT2 expression in HNSCC cell lines (HSC-3, CAL-27, and SAS) and normal epithelial cell lines (HOEC and HIEC6). Data were presented as mean ± standard deviation. ∗∗∗*p* < 0.001 and ∗∗∗∗*p* < 0.0001 for student's *t*-test and one-way ANOVA test. CCT2, chaperonin containing TCP1 subunit 2; HNSCC, head and neck squamous cell carcinoma.

We initially examined the expression of CCT2 in HNSCC tissues. By analyzing five independent datasets (TCGA HNSC, GSE127165, GSE10121, GSE37991, and GSE30784), we identified 311 commonly up-regulated genes (Fig. 1A), with tumor tissues showing significantly higher CCT2 expression compared with adjacent normal tissues across all datasets (Fig. 1B). Univariate Cox regression analysis was conducted to assess the relationship between CCT2 expression and patient prognosis using the GSE41613 and GSE42743 datasets, identifying CCT2 as a common independent risk factor for prognosis (Fig. 1C and D). Kaplan–Meier analysis further revealed that high CCT2 expression was strongly linked to reduced overall survival in HNSCC patients across GSE41613, GSE42743, and TCGA datasets (Fig. 1E).

To validate findings from public databases, we assessed the protein expression of CCT2 and Ki-67 in 41 HNSCC tumor samples and their corresponding adjacent normal tissues via IHC. Our results demonstrated a significant overexpression of CCT2 in HNSCC tissues, with a positive expression rate of 68.29% (28/41), compared with adjacent normal tissues (Fig. 1F and G; Table 1). Additionally, IHC revealed strong co-expression of CCT2 and Ki-67 in tumor tissues, showing a notable positive correlation between their levels (Fig. 1F). Further analysis of clinical factors associated with CCT2 expression revealed that higher CCT2 levels were significantly linked to the clinical stage and TNM classification (*p* = 0.014), with a trend towards correlation with tumor size (*p* = 0.059), but no association with other clinical parameters (Table 2).

**Table 1:** Distribution of CCT2 IHC scores in tumor and adjacent normal tissues.

IHC Score	Tumor Tissue (*n* = 41)	Adjacent Normal Tissue (*n* = 13)	*χ*^*2*^	*p*-value
0	13 (31.7%)	9 (69.2%)	8.88	0.003
1	8 (19.5%)	2 (15.4%)		
2	9 (22.0%)	1 (7.7%)		
3	11 (26.8%)	1 (7.7%)

**Table 2:** Association between immunohistochemical expression of CCT2 and clinicopathological characteristics (*n* = 41).

	CCT2 expression		Significance
	Negative	Positive	*p* value
Age			0.443
≤60(years)	8	22	
>60(years)	5	6	
Gender			0.993
Female	4	7	
Male	9	21	
Tumor size			0.059
T1/T2	12	16	
T3/T4	1	12	
Node category			0.103
N^-^	10	14	
N^+^	3	14	
Pathology differentiation			0.344
Well	4	13	
Moderate/Poor	9	15	
TNM classification			**0.014∗**
I/II	10	10	
III/IV	3	18	
Cigarette			0.756
Yes	9	18	
No	4	10	
Alcohol (>5g/Day)			0.774
Yes	2	7	
No	11	21	
Prognosis			0.608
Well	8	21	
Poor	5	7	

Additionally, Western blotting analysis of tumor and adjacent normal tissues from three HNSCC patients revealed that CCT2 expression was markedly higher in tumor tissues than in adjacent normal tissues (Fig. 1H). Consistent with these results, HNSCC cell lines (HSC-3, CAL-27, and SAS) exhibited significantly higher protein and mRNA levels of CCT2 compared with normal oral squamous epithelial cell lines (HOEC and HIEC6), as demonstrated by Western blotting and quantitative PCR analyses (Fig. 1I and J). These findings indicated that CCT2 was overexpressed in HNSCC and correlated with disease progression as well as a poor prognosis in patients.

### CCT2 knockdown suppressed HNSCC proliferation both *in vitro* and *in vivo*

**Figure 2:**
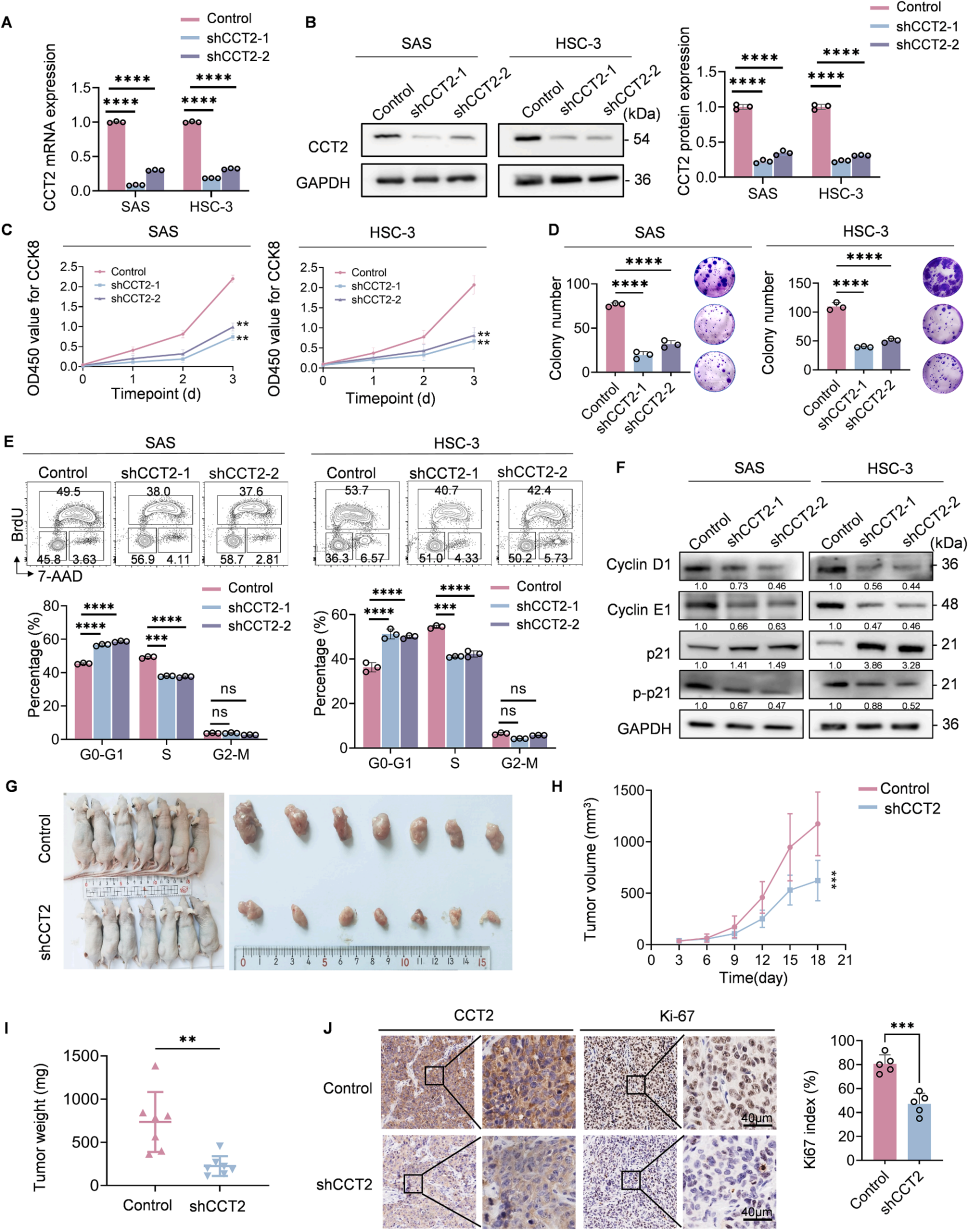
CCT2 knockdown suppressed HNSCC proliferation *in vitro* and *in vivo*. **(A, B)** Quantitative PCR and Western blotting analysis were performed to assess the knockdown efficiency of CCT2 in HSC-3 and SAS cell lines after shRNA-mediated silencing. **(C, D)** CCK-8 assay and colony formation assay were used to evaluate the cell proliferation activity of SAS and HSC-3 cell lines after CCT2 knockdown. **(E)** BrdU incorporation assay was used to evaluate cell proliferation and cell cycle progression in SAS and HSC-3 cell lines following CCT2 knockdown. **(F)** Western blotting analysis of cell cycle-related protein expression in SAS and HSC-3 cell lines. **(G)** Representative images of subcutaneous xenograft tumors on day 21. **(H, I)** Tumor growth curve and tumor weights of nude mice subcutaneously injected with control or shCCT2 SAS cells. **(J)** Representative images of subcutaneous xenograft tumors stained with immunohistochemistry. Scale bar: 40 μm. Data were presented as mean ± standard deviation. ns (not significant), ∗∗*p* < 0.01, ∗∗∗*p* < 0.001, and ∗∗∗∗*p* < 0.0001 for student's *t*-test and one-way ANOVA test. CCT2, chaperonin containing TCP1 subunit 2; HNSCC, head and neck squamous cell carcinoma.

To explore the role of CCT2 in HNSCC progression, we selected HSC-3 cells with the highest endogenous CCT2 expression and SAS cells with the lowest expression for subsequent experiments (Fig. 1I and J). CCT2 expression was knocked down in both cell lines using shRNA, which was validated by Western blotting and quantitative PCR (Fig. 2A and B).

CCK-8 and colony formation assays demonstrated that CCT2 knockdown significantly suppressed the proliferation and colony-forming abilities of both HSC-3 and SAS cells (Fig. 2C and D), suggesting that CCT2 was essential in driving the growth of HNSCC cells. Given the role of the cell cycle in regulating proliferation, BrdU incorporation assays were performed and the results revealed that knockdown of CCT2 notably elevated the percentage of cells in the G0/G1 phase, while decreasing the proportion in the S phase in both cell lines (Fig. 2E). Consistently, Western blotting analysis demonstrated that CCT2 knockdown significantly down-regulated the expression of cyclin D1 (CCND1), cyclin E1 (CCNE1), and phospho-p21 (p-p21), while up-regulating total p21 levels (Fig. 2F), suggesting that CCT2 depletion induces G1/S phase cell cycle arrest through modulation of these key regulators.

To validate these findings *in vivo*, we subcutaneously injected 1 × 10^6^ SAS cells (control and shCCT2) into nude mice and measured tumor volumes every three days. By day 21 post-injection, the mice were sacrificed, and tumor sizes were analyzed. Tumors derived from shCCT2 SAS cells were significantly smaller compared with controls, and the tumor growth curve showed a markedly reduced tumor growth rate in the shCCT2 group (Fig. 2G–I). IHC analysis further revealed that the expression of Ki-67 was markedly reduced in tumors derived from shCCT2 cells (Fig. 2J), consistent with the *in vitro* findings. Together, these results demonstrate that CCT2 enhances HNSCC cell proliferation and tumor growth by modulating cell cycle progression.

### CCT2 knockdown inhibits HNSCC cell migration and invasion by suppressing epithelial–mesenchymal transition

**Figure 3:**
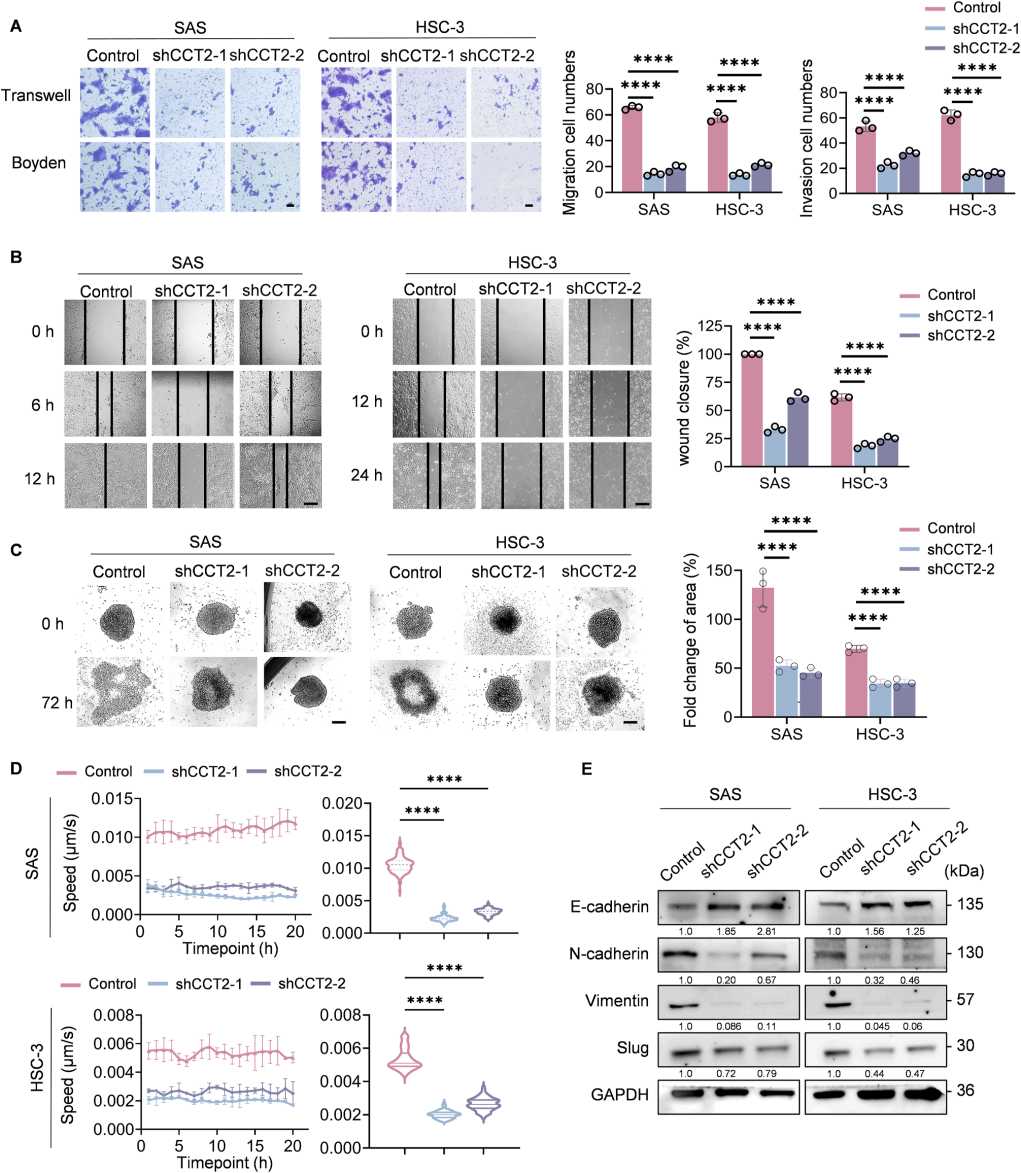
CCT2 knockdown inhibited the invasion and migration of HNSCC. **(A)** The representative images and the quantitative data of Transwell and Boyden assays in SAS and HSC-3 cells. Scale bar: 100 μm. **(B)** Wound healing assay in SAS and HSC-3 cells after CCT2 knockdown. Scale bar: 400 μm. **(C)** Representative images and statistical graphs of migration area analysis of tumor sphere 72 h after embedding in SAS and HSC-3 cells. Scale bar: 100 μm. **(D)** Single cell motility assay of SAS and HSC-3 cells for 20 h. **(E)** Western blotting analysis of epithelial–mesenchymal transition (EMT)-related protein expression in SAS and HSC-3 cell lines. Data were presented as mean ± standard deviation. ns (not significant) and ∗∗∗∗*p* < 0.0001 for student's *t*-test and one-way ANOVA test. CCT2, chaperonin containing TCP1 subunit 2; HNSCC, head and neck squamous cell carcinoma.

To explore whether CCT2 knockdown affected the migration and invasion capabilities of HNSCC cells, transwell and Boyden assays were conducted (Fig. 3A). The migration and invasion capabilities were notably decreased in SAS and HSC-3 cells transfected with CCT2-shRNA when compared with control cells. To validate these findings, we performed a wound healing assay (Fig. 3B) to monitor cell migration over time. CCT2 knockdown delayed wound closure in SAS and HSC-3 cells, indicating impaired migratory capacity.

We further used a tumor sphere migration assay (Fig. 3C) to assess collective cell migration. After 72 h, the migration areas of tumor spheres were markedly reduced in CCT2-knockdown SAS and HSC-3 cells compared with controls. This 3D model closely mimics the tumor microenvironment, underscoring the significance of CCT2 in facilitating collective tumor cell movement under physiologically relevant conditions. In addition, a single-cell motility assay (Fig. 3D) revealed a clear reduction in motility and distance traveled by CCT2-knockdown cells, confirming the role of CCT2 in promoting individual cell motility.

Epithelial–mesenchymal transition is a critical event in tumor metastasis, allowing epithelial cells to adopt mesenchymal characteristics, which promotes migration and invasion.^28^ Knockdown of CCT2 led to an increase in the expression of the epithelial marker E-cadherin and a decrease in mesenchymal markers, including N-cadherin, vimentin, and Slug (Fig. 3E). These findings indicate that CCT2 depletion suppresses epithelial–mesenchymal transition, contributing to the observed reduction in migratory and invasive capacities of HNSCC cells.

### CCT2 knockdown enhanced cisplatin sensitivity in HNSCC

**Figure 4:**
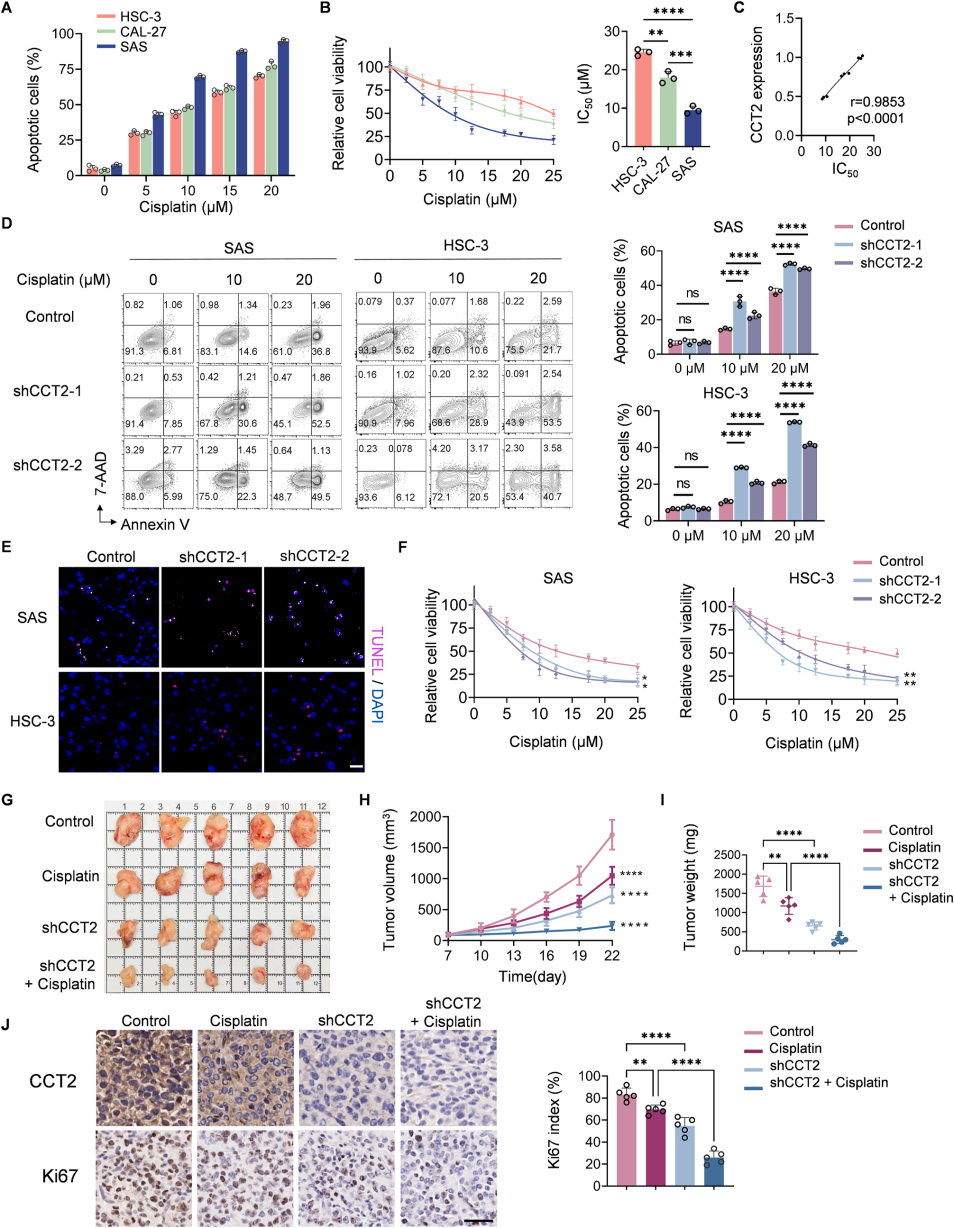
CCT2 plays a pivotal role in mediating cisplatin resistance in HNSCC. **(A)** Quantification of apoptosis of SAS, HSC-3, and CAL-27 cell lines treated with cisplatin for 48 h at varying concentrations by flow cytometric analysis of annexin-V staining and 7AAD staining. **(B)** CCK-8 assays were performed to determine the resistance or sensitivity to cisplatin in SAS, CAL-27, and HSC-3 cell lines, and IC_50_ was calculated. **(C)** The relationship between CCT2 expression and IC_50_ in three HNSCC cell lines. **(D)** Apoptosis rate of SAS and HSC-3 cell lines with or without CCT2 knockdown, treated with 20 μM cisplatin for 24 h. **(E)** Representative images of TUNEL staining in SAS and HSC-3 cell lines with lentivirus infection, treated with cisplatin. Scale bar: 25 μm. **(F)** CCK8 assay was used to assess the sensitivity to cisplatin after CCT2 silencing in SAS and HSC-3 cell lines. **(G)** Representative images of subcutaneous xenograft tumors on day 22. **(H, I)** Tumor growth curves and tumor weight of C57BL/6 mice injected subcutaneously with MOC1 control or CCT2-knockdown cells. **(J)** Immunohistochemistry images of subcutaneous xenograft tumors. Scale bar: 40 μm. Data were presented as mean ± standard deviation. ns (not significant), ∗∗*p* < 0.01, ∗∗∗*p* < 0.001, and ∗∗∗∗*p* < 0.0001 for student's *t*-test and one-way ANOVA test. CCT2, chaperonin containing TCP1 subunit 2; HNSCC, head and neck squamous cell carcinoma.

Cisplatin is a cornerstone chemotherapeutic agent in the treatment of HNSCC, but resistance to cisplatin is a major clinical challenge, limiting its effectiveness.^29^ We tested three HNSCC cell lines and observed that CCT2 expression was most elevated in HSC-3 cells, followed by CAL-27, and lowest in SAS cells (Fig. 1F). To determine the potential link between CCT2 and cisplatin resistance, we treated these cell lines with varying concentrations of cisplatin. Apoptosis analysis (Fig. 4A) showed a dose-dependent increase in apoptosis. Using CCK-8 assays (Fig. 4B), IC_50_ values were calculated, revealing the highest IC50 in HSC-3, followed by CAL-27 and SAS. This stepwise trend strongly correlated with CCT2 expression (*r* = 0.9853, *p* < 0.0001; Fig. 4C), linking higher CCT2 levels to greater resistance.

To further investigate whether CCT2 influenced cisplatin resistance in HNSCC, we performed flow cytometry analysis (Fig. 4D) and demonstrated that CCT2 knockdown significantly increased the sensitivity of SAS and HSC-3 cells to cisplatin, as evidenced by elevated apoptosis rates. Interestingly, this effect was more pronounced in HSC-3 cells, which have higher endogenous CCT2 expression than SAS cells. TUNEL staining (Fig. 4E) confirmed increased DNA fragmentation in CCT2-knockdown cells treated with cisplatin. CCK-8 assays (Fig. 4F) showed that reduced CCT2 expression caused a shift of the dose–response curve downward towards cisplatin, suggesting increased sensitivity.

*In vivo*, MOC1 cells, a mouse-derived oral squamous cell carcinoma cell line, were used to establish xenograft models in C57BL/6 mice. Control and CCT2-knockdown MOC1 cells were implanted into mice that received cisplatin or PBS intraperitoneally starting on day 7. Subcutaneous xenograft tumor images (Fig. 4G) and tumor growth curves (Fig. 4H) showed that cisplatin significantly reduced tumor volume in the CCT2-knockdown group. Tumor weight measurements (Fig. 4I) confirmed that the combination (shCCT2 + cisplatin) group had the lowest tumor weight. IHC analysis (Fig. 4J) showed reduced CCT2 and Ki-67 expression in CCT2-knockdown tumors, and their expression further diminished with cisplatin treatment. These findings highlight that CCT2 knockdown enhances cisplatin sensitivity in HNSCC both *in vitro* and *in vivo*.

### CCT2 stabilized c-Myc to drive cell cycle progression and cisplatin resistance in HNSCC

**Figure 5:**
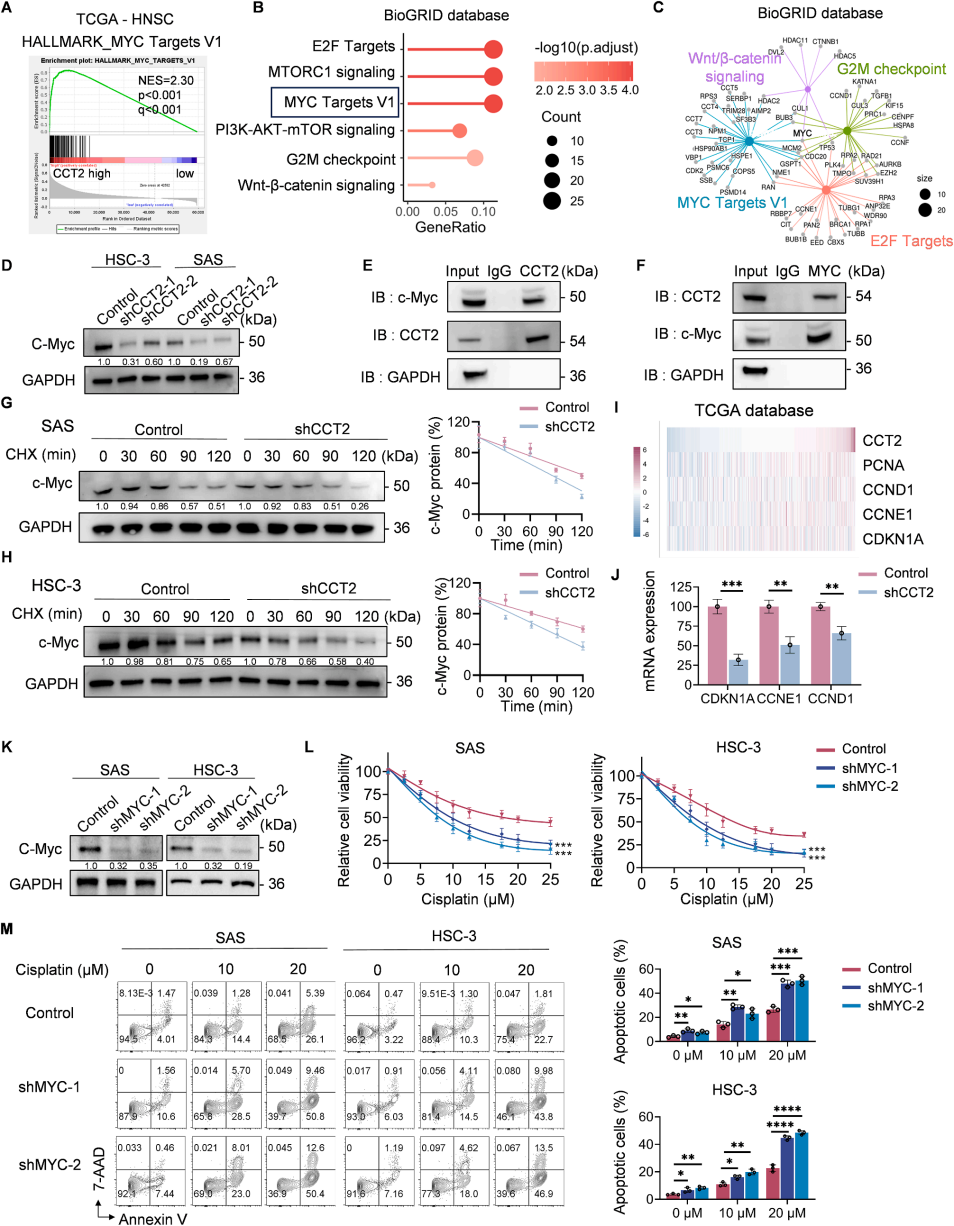
CCT2 inhibition destabilizes c-Myc and impairs both its transcriptional activity and cisplatin resistance capacity. **(A)** GSEA analysis of the TCGA HNSCC cohort shows significant enrichment of the MYC Target_V1 pathway in patients with high CCT2 expression. **(B)** Hallmark enrichment analysis of identified interaction proteins with CCT2 in the BioGRID Database. **(C)** Network plot of cell cycle-related pathways enriched in CCT2-interacting proteins, with genes representing each pathway. **(D)** The expression level of c-Myc protein decreased due to CCT2 knockdown in HSC-3 and SAS cells. **(E, F)** The interaction between CCT2 and c-Myc was tested with co-immunoprecipitation in HSC-3 cells. **(G, H)** Time-course analysis of c-Myc expression in SAS and HSC-3 cells with or without CCT2 depletion. Cells were treated with cycloheximide (100 μg/mL) for varying periods. **(I)** Heatmap of cell cycle-related gene expression in the TCGA HNSCC cohort, ordered by CCT2 expression from low to high. **(J)** Quantitative PCR analysis of the mRNA expression of CCND1, CCNE1, and CDKN1A in HSC-3 cells. **(K)** Western blotting analysis of c-Myc protein levels in SAS and HSC-3 cells after transfection with c-Myc-targeting shRNA. **(L)** CCK8 assay was used to assess the sensitivity to cisplatin after c-Myc silencing in SAS and HSC-3 cell lines treated with different concentrations of cisplatin. **(M)** Apoptosis rate of SAS and HSC-3 cell lines with or without c-Myc knockdown, treated with 10 μM or 20 μM cisplatin for 24 h. Data were presented as mean ± standard deviation. ∗∗*p* < 0.01 and ∗∗∗*p* < 0.001 for student's *t*-test and one-way ANOVA test. CCT2, chaperonin containing TCP1 subunit 2; HNSCC, head and neck squamous cell carcinoma; CCND1, cyclin D1; CCNE1, cyclin E1; CDKN1A, cyclin-dependent kinase inhibitor 1A.

To further investigate the molecular mechanisms underlying the role of CCT2 in HNSCC aggressiveness and drug resistance, we divided the HNSC cohort in the TCGA database into two groups based on CCT2 expression (high *vs*. low) and performed GSEA enrichment analysis, revealing significant enrichment of the MYC Target_V1 pathway in the high CCT2 expression group (Fig. 5A). We conducted an enrichment analysis of proteins interacting with CCT2, identified from the BioGRID database, using the Hallmark gene set. The results revealed significant enrichment of cell cycle-related pathways, including c-Myc and other key regulators of cell cycle progression (Fig. 5B). To visualize these interactions, we constructed an interaction map (Fig. 5C) highlighting c-Myc as a central node within several enriched pathways.

To validate the previous findings, we investigated the impact of CCT2 on c-Myc expression in the HSC-3 and SAS cell lines. Knockdown of CCT2 in both cell lines resulted in a significant decrease in c-Myc protein levels (Fig. 5D). To further explore whether CCT2 regulated c-Myc stability, we conducted co-immunoprecipitation experiments in HSC-3 cells, which confirmed a direct interaction between CCT2 and c-Myc (Fig. 5E and F). Cycloheximide treatment was used to assess c-Myc degradation in SAS and HSC3 cells with or without CCT2 knockdown. Cycloheximide treatment was used to assess c-Myc degradation in SAS and HSC3 cells with or without CCT2 knockdown. The results indicated that c-Myc stability was better in control cells than in shCCT2 cells, with a significantly reduced c-Myc level and shorter half-life in shCCT2 cells after cycloheximide treatment (Fig. 5G and H).

Furthermore, we analyzed data from the TCGA database, ordering the cohort based on CCT2 expression levels from low to high. A heatmap revealed a positive correlation between CCT2 expression and key cell cycle-related proteins, including proliferating cell nuclear antigen (PCNA), CCND1, CCNE1, and cyclin-dependent kinase inhibitor 1A (CDKN1A) (Fig. 5I). To validate these findings, quantitative PCR analysis was performed in HSC-3 cells, showing that knockdown of CCT2 significantly reduced the mRNA expression levels of CCND1, CCNE1, and CDKN1A, consistent with the TCGA database results (Fig. 5J). To determine whether CCT2-mediated regulation of c-Myc influenced cisplatin resistance in HNSCC cells, we knocked down c-Myc expression in SAS and HSC-3 cells using shRNA (Fig. 5K). CCK-8 and flow cytometry assays demonstrated that c-Myc knockdown significantly sensitized both cell lines to cisplatin treatment, indicating that c-Myc plays a critical role in mediating cisplatin resistance in HNSCC (Fig. 5L and M). These results demonstrate that CCT2 stabilizes c-Myc while up-regulating cell cycle-related molecules and enhancing cisplatin resistance in HNSCC.

### c-Myc overexpression rescues CCT2-knockdown HNSCC cells from cycle arrest, migration impairment, and drug resistance

**Figure 6:**
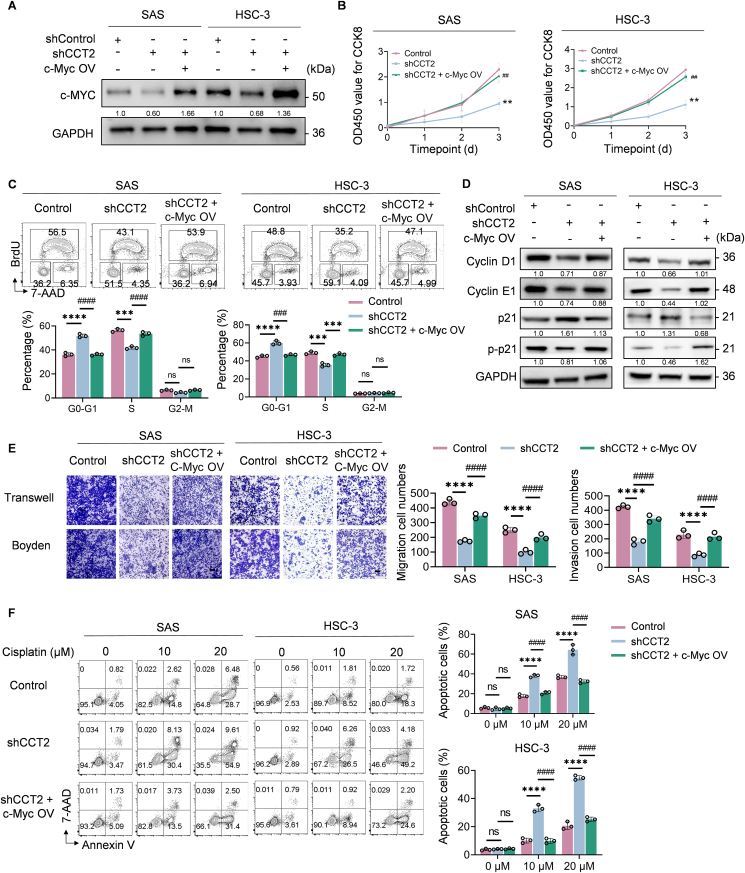
c-Myc is a functional downstream target of CCT2. **(A)** Western blot images of the c-Myc protein level after c-Myc overexpression in CCT2-knockout cells. **(B)** CCK8 analysis of SAS and HSC-3 cells with indicated modifications. **(C)** BrdU assay verified the effect of c-Myc overexpression on the cell cycle. **(D)** Western blot images of CCND1, CCNE1, p21, and p-p21 protein levels after c-Myc overexpression in CCT2-knockout cells. **(E)** Transwell and Boyden chamber assays were used to assess the effect of c-Myc rescue after CCT2 knockout on cell migration and invasion abilities. Scale bar: 100 μm. **(F)** Apoptosis rate of SAS and HSC-3 cells with indicated treatments. Data were presented as mean ± standard deviation. ns (not significant), ∗∗*p* < 0.01, ∗∗∗*p* < 0.001, and ∗∗∗∗*p* < 0.0001 for student's *t*-test and one-way ANOVA test compared with the control group. ^##^*p* < 0.01, ^###^*p* < 0.001, and ^####^*p* < 0.0001 for student's *t*-test and one-way ANOVA test compared with the shCCT2 group. CCT2, chaperonin containing TCP1 subunit 2; CCND1, cyclin D1; CCNE1, cyclin E1.

To further investigate whether c-Myc was a functional downstream molecule of CCT2, we overexpressed c-Myc in CCT2 knockdown SAS and HSC-3 cells (Fig. 6A). The CCK-8 assay revealed that c-Myc overexpression nearly completely rescued both SAS and HSC-3 cells from the proliferation inhibition caused by CCT2 knockdown (Fig. 6B). BrdU incorporation assays also confirmed that c-Myc overexpression alleviated the G1/S phase cell cycle arrest induced by CCT2 depletion (Fig. 6C), with elevated levels of critical cell cycle-promoting proteins (Fig. 6D).

In addition to promoting cell proliferation, Transwell and Boyden chamber assays demonstrated that the increased expression of c-Myc restored the impaired migration and invasion abilities of the shCCT2 cells (Fig. 6E). Notably, c-Myc overexpression also reestablished resistance to cisplatin in shCCT2 cells, a phenomenon that closely mirrors clinical observations^30^ (Fig. 6F). These findings collectively suggest that c-Myc is a critical mediator of CCT2's effects on cell proliferation, migration, invasion, and drug resistance in HNSCC cells.

apoVs promoted cisplatin resistance in recipient tumor cells by transferring CCT2.

**Figure 7:**
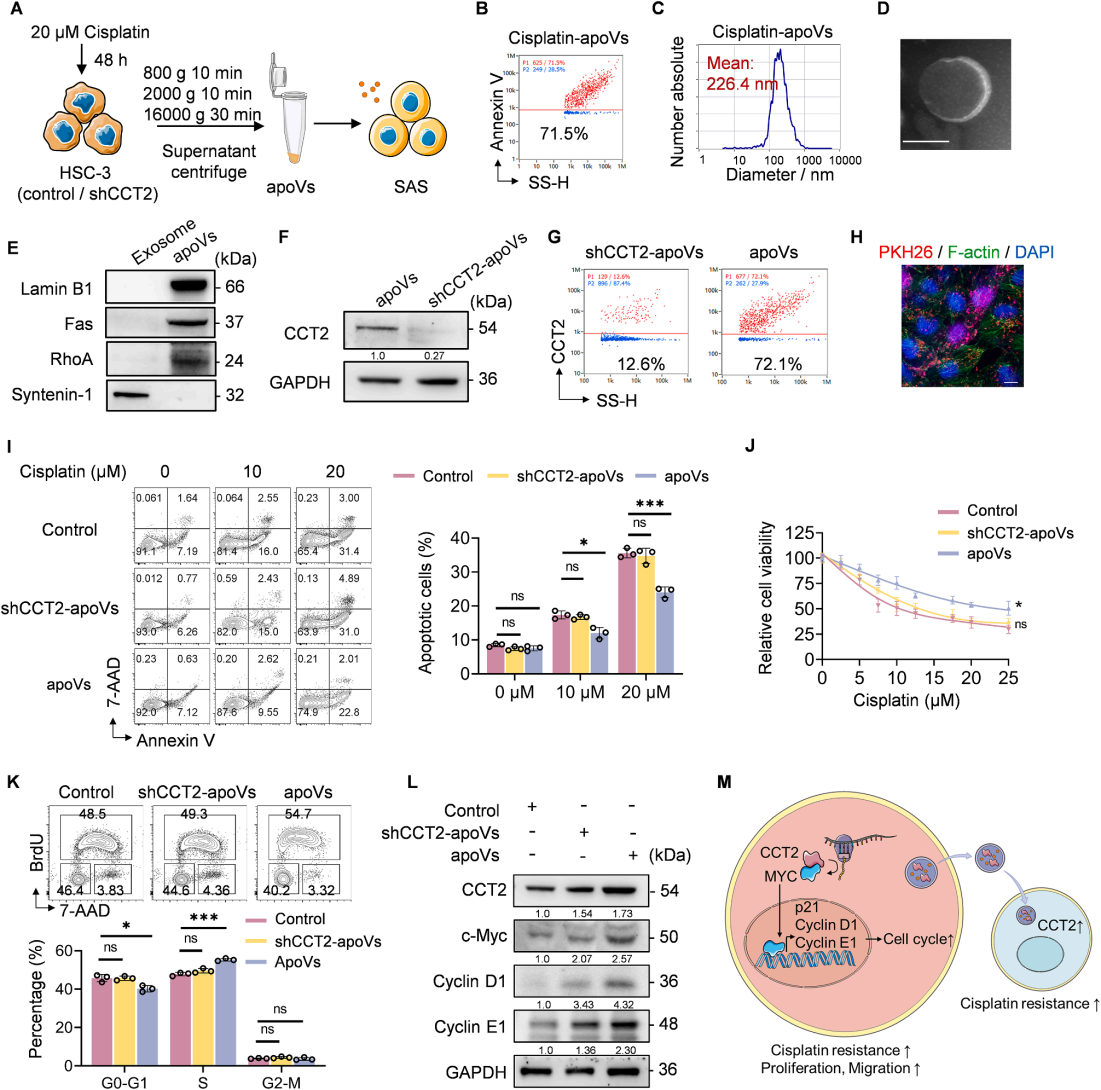
apoVs from HSC-3 cells transfer CCT2 to SAS cells and promote their cisplatin resistance. **(A)** HSC-3 control and shCCT2 cells were treated with 20 μM cisplatin for 48 h to induce apoptosis. The apoptotic cell suspensions were then isolated using a sequential centrifugation system to obtain apoVs. **(B)** Nanoflow cytometry analysis showed the annexin V-positive rate of apoVs. **(C)** Nanoparticle tracking analysis confirmed the size distribution of apoVs. **(D)** Transmission electron microscopy assay showed the morphology of apoVs. Scale bar: 200 nm. **(E)** Western blotting revealed the expression of lamin B1, FAS, RhoA, and syntenin-1 in aopVs and exosomes. **(F)** Western blot images of CCT2 expression in apoVs derived from control and shCCT2 HSC-3 cells. **(G)** Nanoflow cytometry analysis showed the positive rate of CCT2 in apoVs. **(H)** Immunofluorescence images showed that the apoVs were internalized by receptor-tumor cells. Scale bar: 10 μm. **(I)** Flow cytometry analysis showed the apoptosis levels of SAS cells after uptake of apoVs from HSC-3 control and shCCT2 cells, followed by treatment with different concentrations of cisplatin for 2 days. **(J)** CCK-8 assay evaluated the effect of apoVs on the cisplatin sensitivity of SAS cells. **(K)** BrdU assay confirmed that apoVs influenced the cell cycle of SAS cells. **(L)** Western blotting assay showed that apoVs affected the expression levels of CCT2, c-Myc, CCND1, and CCNE1 proteins in SAS cells. **(M)** Schematic diagram of CCT2 stabilizing c-Myc to drive tumor progression and chemoresistance in HNSCC, with vesicle-mediated transfer promoting drug resistance in recipient cells. Data were presented as mean ± standard deviation. ns (not significant), ∗*p* < 0.05 and ∗∗∗*p* < 0.001 for student's *t*-test and one-way ANOVA test. apoVs, apoptotic vesicles; CCT2, chaperonin containing TCP1 subunit 2; CCND1, cyclin D1; CCNE1, cyclin E1; HNSCC, head and neck squamous cell carcinoma.

Chemotherapy-induced acquired resistance poses a major challenge in clinical cancer treatment, with extracellular vehicles increasingly recognized as critical players in this process.^24^ To investigate whether apoVs derived from drug-resistant tumor cells promoted resistance in recipient tumor cells, we isolated apoVs from HSC-3 control and shCCT2 cells treated with 20 μM cisplatin for 48 h, following the workflow shown in Fig. 7A. Nanoflow cytometry revealed that the apoVs were 71.5% annexin V positive, with an average diameter of 226.4 nm, and electron microscopy confirmed their normal structure (Fig. 7B–D). Compared with exosomes, apoVs exhibited higher expression of lamin B1, Fas, and RhoA, while the exosome-specific marker syntenin-1 was barely detectable (Fig. 7E). Western blotting analysis (Fig. 7F) and nanoflow cytometry (Fig. 7H) demonstrated that apoVs derived from control HSC-3 cells had significantly higher levels of CCT2 compared with those from shCCT2 cells, and these apoVs could be internalized by recipient SAS cells (Fig. 7H).

Treatment of SAS cells with apoVs derived from HSC-3 control cells significantly enhanced cisplatin resistance, while apoVs from shCCT2 cells showed a markedly reduced ability to promote resistance (Fig. 7I). CCK-8 assays further confirmed the importance of CCT2 in apoVs-mediated enhancement of cisplatin resistance in recipient cells (Fig. 7J). Given that both CCT2 and c-Myc are closely associated with cell cycle regulation, a key mechanism underlying cisplatin resistance, we conducted BrdU incorporation assays. The results revealed that apoVs notably expedited cell cycle progression in SAS cells, while also up-regulating the expression of c-Myc and other key cell cycle proteins, including CCND1 and CCNE1 (Fig. 7K and L). In contrast, apoVs derived from shCCT2 cells had minimal effects on cell cycle progression, explaining their inability to enhance cisplatin resistance. These findings suggest that CCT2 transmitted via apoVs from drug-resistant tumor cells may promote cisplatin resistance in recipient tumor cells by accelerating cell cycle progression.

## Discussion

Rapid progression and resistance during treatment pose significant challenges in HNSCC therapy, with the underlying drivers remaining unclear. The CCT family contributes to tumor progression through multiple mechanisms, with its subunits influencing the regulation of several key cell-cycle proteins. TRiC/CCT is crucial for the correct folding of CDC20, making it vital for CDC20-dependent cell cycle processes, including sister chromatid separation and checkpoint exit during mitosis.^31^ Cytoskeletal proteins, including tubulin and actin, are also mandatory substrates for TRiC/CCT folding. CCT8, in particular, enhances glioma cell migration and invasion by controlling cytoskeletal reorganization.^32^ Further studies have shown that CCT subunits in tumor cells play a role in regulating glucose and lipid metabolism reprogramming through both transcriptional and signaling pathways.^33^ Our research revealed that CCT2 was overexpressed in HNSCC, facilitating cell proliferation, migration, and invasion. It was also linked to poor patient prognosis, supporting the role of CCT2 as a key driver of HNSCC.

Protein homeostasis not only provides a foundation for the rapid proliferation and metastasis of tumor cells but also is closely associated with tumor drug resistance. The link between the CCT family and drug resistance has garnered increasing attention. CCT3 increases ferroptosis resistance in liver cancer cells by inhibiting iron endocytosis via α-actinin-4 (ACTN4)/transferrin receptor (TFRC).^34^ Mass spectrometry analysis has identified CCT2 as involved in multi-drug resistance in lung cancer cells, such as DLKP.^35^ TCP1 enhances resistance to acute myeloid leukemia by inhibiting autophagy through the activation of the protein kinase B (AKT)/mechanistic target of rapamycin (mTOR) signaling pathway.^12^ Consistently, here, we also provided the first evidence that CCT2 induced cisplatin resistance by stabilizing c-Myc to regulate cell cycle progression in HNSCC.

c-Myc is a classic oncogene that is aberrantly activated or overexpressed in many human cancers. It promotes tumor cell proliferation and growth by modulating the expression of cell cycle-associated proteins, including CDKs and cyclins, thereby promoting the rapid transition of tumor cells into the S phase.^36^ Furthermore, c-Myc drives chemoresistance across diverse cancers (*e.g.*, diffuse large B-cell lymphoma/R–CHOP (rituximab, cyclophosphamide, doxorubicin, vincristine, and prednisone),^37^ breast cancer/doxorubicin,^38^ colorectal cancer/5-fluorouracil,^39^ non-small cell lung cancer/cisplatin^40^) via metabolic reprogramming, enhanced DNA repair, apoptosis suppression, and drug efflux.^41^, ^42^, ^43^ Despite its critical role in tumor development and progression, directly targeting c-Myc remains a significant challenge due to the lack of specific binding sites and its predominant nuclear localization.^19^ Targeting CCT2, which stabilizes c-Myc protein, emerges as a promising therapeutic strategy. The CCT inhibitor CT20p has already shown therapeutic efficacy in breast cancer,^44^ suggesting the potential of CCT family inhibitors in cancer treatment. However, further drug development and experimental evidence are needed to explore the feasibility of targeting CCT2 as a therapeutic approach for HNSCC.^45^

Our study further revealed that CCT2 not only promoted drug resistance in tumor cells but also mediated the non-genetic plasticity of recipient tumor cells through the transfer of apoVs, leading to drug tolerance. Recent studies suggest that adaptive resistance in cancer treatment is not solely driven by genetic mutations; non-genetic factors can induce transient drug tolerance, leading to cellular state transitions and, over time, the acquisition of long-term phenotypic plasticity.^46^ Tumor therapies, such as chemotherapy and radiotherapy, induce massive apoptosis, resulting in a substantial number of apoVs being released into the circulatory system and tumor microenvironment.^47^ These apoVs have been shown to enhance glioblastoma malignancy,^22^ modulate macrophage function,^48^ and promote angiogenesis,^49^ thereby remodeling the tumor microenvironment. This evidence reveals that apoVs may play a crucial role in the adaptive resistance of residual tumor cells. In this study, we found that CCT2 carried by apoVs accelerates the cell cycle of recipient tumor cells, thereby conferring cisplatin resistance. This provides a novel explanation for chemotherapy-driven tumor evolution and highlights the vital therapeutic potential of targeting CCT2 in the treatment of HNSCC. These findings emphasize the importance of investigating apoVs as mediators of tumor adaptability and resistance, further reinforcing the potential of CCT2 as a valuable therapeutic target for addressing drug resistance in cancer.

## Conclusion

In summary, our study underscores the pivotal role of CCT2 in the progression, metastasis, and cisplatin resistance of HNSCC. We demonstrated that CCT2 was overexpressed in HNSCC and linked to worse prognosis, advanced clinical stages, and more aggressive tumor characteristics. Functionally, CCT2 enhanced the proliferation, migration, and invasion of HNSCC cells, as well as their resistance to cisplatin, by stabilizing c-Myc and influencing critical cell cycle regulators. Moreover, apoVs from drug-resistant tumor cells were shown to enhance cisplatin resistance in recipient tumor cells via transferring CCT2, further amplifying its role in chemoresistance (Fig. 7M). These findings identify CCT2 as a promising therapeutic target to improve HNSCC outcomes and overcome drug resistance, emphasizing the need for further exploration of its molecular mechanisms and therapeutic capabilities.

## CRediT authorship contribution statement

**Lin Lu:** Conceptualization, Writing – original draft, Investigation, Methodology. **Yi Shen:** Investigation, Methodology, Software, Conceptualization. **Xuerong Li:** Investigation, Software. **Yiwei Zhao:** Validation, Data curation. **Xuefan Zhai:** Investigation, Visualization. **Min Cai:** Resources, Methodology. **Baicheng Bao:** Supervision. **Guiqing Liao:** Supervision, Resources, Writing – review & editing, Conceptualization. **Jianbo Sun:** Supervision, Writing – review & editing, Conceptualization.

## Ethics declaration

All animal experiments followed institutional and national guidelines, adhering to ARRIVE guidelines, and were approved by the Institutional Animal Care and Use Committee at Sun Yat-sen University (SYSU-IACUC-2020-000584 & SYSU-IACUC-2022-000027). Informed consent was obtained from all participants or their legal guardians. The study was conducted in accordance with relevant regulations and approved by the Medical Ethics Committee of the Hospital of Stomatology, Sun Yat-sen University (KQEC-2021-76-01).

## Data availability

The datasets generated or analyzed during this study are available upon reasonable request.

## Funding

This work is supported by the 10.13039/501100001809National Natural Science Foundation of China (No. 81672679, 82103558 to Guiqing Liao), Dongguan Science and Technology of Social Development Program (Guangdong, China) (No. 20221800905652to Jianbo Sun), 10.13039/501100021171Guangdong Basic and Applied Basic Research Foundation (China) (No. 2022A1515010678, 2022A1515140101, 2021A1515012324 to Jianbo Sun), Clinical plus Basic Innovation Project of Guangdong Medical University (China) (No. 4SG24012G to Jianbo Sun), Talent Development Foundation of The First Dongguan Affiliated Hospital of Guangdong Medical University (China) (No. GCC2022003 to Jianbo Sun), and the Basic and Applied Basic Research Foundation of Guangzhou City, Guangdong, China (No. 202002030127 to Jianbo Sun).

## Conflict of interests

The authors declared no competing interests.
