# Supplementary material for: CCT2 enhances c-Myc stabilization to drive tumor progression and chemoresistance in head and neck squamous cell carcinoma
Source: Genes Dis. 2025 Aug 7;13(6):101795. doi: 10.1016/j.gendis.2025.101795 (PMC13485502; doi:10.1016/j.gendis.2025.101795)
Supplement: Supplementary file 1 [file mmc1.docx]

**Supplementary Table S1 List of Primary Antibodies Used In the study**

| Antibody | Company | Category |
| --- | --- | --- |
| CCT2 | Santa Cruz | sc-374152 |
| Cyclin D1 | CST | 2978 |
| Cyclin E1 | Proteintech | 11554-1-AP |
| p21 | Santa Cruz | sc-6246 |
| p-p21 | Santa Cruz | sc-377514 |
| c-Myc | Proteintech | 10828-1-AP |
| GAPDH | Proteintech | 60004-1-Ig |
| Lamin B1 | Abcam | ab133741 |
| FAS | Santa Cruz | sc-74540 |
| RhoA | Santa Cruz | sc-418 |
| syntenin-1 | Abcam | ab19903 |
