# Supplementary material for: CCT2 enhances c-Myc stabilization to drive tumor progression and chemoresistance in head and neck squamous cell carcinoma
Source: Genes Dis. 2025 Aug 7;13(6):101795. doi: 10.1016/j.gendis.2025.101795 (PMC13485502; doi:10.1016/j.gendis.2025.101795)
Supplement: Supplementary file 2 [file mmc2.docx]

**Supplementary Table S2. Sequences of Primer for Real-time Polymerase Chain Reaction**

| **CCT2** |  |
| --- | --- |
| Forward | 5’-GAACGCCTAGCTCTTGTCACA-3’ |
| Reverse | 5’-GCACCACGCAAAACAATGGTA-3’ |
| **GAPDH** |  |
| Forward | 5’-GGTATGACAACGAATTTGGC-3’ |
| Reverse | 5’-GAGCACAGGGTACTTTATTG-3’ |
| **CCNE1** |  |
| Forward | 5’- AAGGAGCGGGACACCATGA-3’ |
| Reverse | 5’- ACGGTCACGTTTGCCTTCC-3’ |
| **CDKN1A** |  |
| Forward | 5’- TGTCCGTCAGAACCCATGC -3’ |
| Reverse | 5’- AAAGTCGAAGTTCCATCGCTC -3’ |
| **MYC** |  |
| Forward | 5’- GGCTCCTGGCAAAAGGTCA -3’ |
| Reverse | 5’- CTGCGTAGTTGTGCTGATGT -3’ |
| **CCND1** |  |
| Forward | 5’-GCTGCGAAGTGGAAACCATC-3’ |
| Reverse | 5’-CCTCCTTCTGCACACATTTGAA-3’ |
